# CSV2018: The 2nd Symposium of the Canadian Society for Virology

**DOI:** 10.3390/v11010079

**Published:** 2019-01-18

**Authors:** Nathalie Grandvaux, Craig McCormick

**Affiliations:** 1 Département de Biochimie et Médecine Moléculaire, Université de Montréal, QC H3C 3J7, Canada; 2 Centre de Recherche du CHUM (CRCHUM), Montréal, QC H2X 0A9, Canada; 3 Department of Microbiology and Immunology, Dalhousie University, 5850 College Street, Halifax, NS B3H 4R2, Canada

**Keywords:** Canada, virology, CSV, influenza, CRISPR, RSV, synthetic virology

## Abstract

The 2nd Symposium of the Canadian Society for Virology (CSV2018) was held in June 2018 in Halifax, Nova Scotia, Canada, as a featured event marking the 200th anniversary of Dalhousie University. CSV2018 attracted 175 attendees from across Canada and around the world, more than double the number that attended the first CSV symposium two years earlier. CSV2018 provided a forum to discuss a wide range of topics in virology including human, veterinary, plant, and microbial pathogens. Invited keynote speakers included David Kelvin (Dalhousie University and Shantou University Medical College) who provided a historical perspective on influenza on the 100th anniversary of the 1918 pandemic; Sylvain Moineau (Université Laval) who described CRISPR-Cas systems and anti-CRISPR proteins in warfare between bacteriophages and their host microbes; and Kate O’Brien (then from Johns Hopkins University, now relocated to the World Health Organization where she is Director of Immunization, Vaccines and Biologicals), who discussed the underlying viral etiology for pneumonia in the developing world, and the evidence for respiratory syncytial virus (RSV) as a primary cause. Reflecting a strong commitment of Canadian virologists to science communication, CSV2018 featured the launch of Halifax’s first annual Soapbox Science event to enable public engagement with female scientists, and the live-taping of the 499th episode of the This Week in Virology (TWIV) podcast, hosted by Vincent Racaniello (Columbia University) and science writer Alan Dove. TWIV featured interviews of CSV co-founders Nathalie Grandvaux (Université de Montréal) and Craig McCormick (Dalhousie University), who discussed the origins and objectives of the new society; Ryan Noyce (University of Alberta), who discussed technical and ethical considerations of synthetic virology; and Kate O’Brien, who discussed vaccines and global health. Finally, because CSV seeks to provide a better future for the next generation of Canadian virologists, the symposium featured a large number of oral and poster presentations from trainees and closed with the awarding of presentation prizes to trainees, followed by a tour of the Halifax Citadel National Historic Site and an evening of entertainment at the historic Alexander Keith’s Brewery.

## 1. Introduction

The Canadian Society for Virology (CSV) was founded in 2016 to provide support for the Canadian virology research community, including basic, clinical, government, and industry researchers working on a broad range of viruses. To launch the new society, CSV planned a satellite workshop in conjunction with the annual American Society for Virology (ASV) meeting in Blacksburg, Virginia in June 2016, which was supported by then-ASV president and ex-pat Canadian Dr. Grant McFadden [1]. Exit surveys expressed strong support for the new society and a desire for additional symposia and other supports for the virology community. Galvanized by this successful event, planning began for the second symposium, this time to be held on Canadian soil at Dalhousie University in Halifax, Nova Scotia, to coincide with the 200th anniversary of the university. Guiding principles during symposium planning included ensuring gender parity across the board, from the Scientific Advisory Committee to the Local Organizing Committee to the invited speakers and trainee speakers selected from abstracts. As at the first symposium, trainee presentations outnumbered presentations from principal investigators, and a large fund was secured for travel awards and oral and poster presentation awards, all earmarked for trainees.

**Figure 1:**
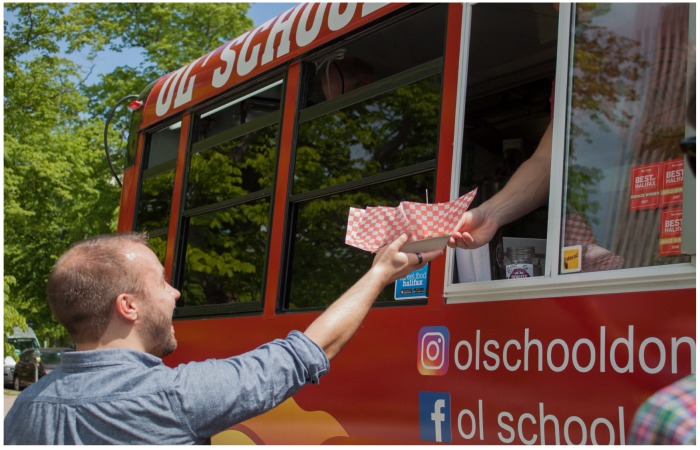
**Who doesn’t love doughnuts?** CSV2018 volunteer Lucas Jarche (who designed the CSV logo) demonstrates proper form in receiving a piping-hot order of fresh doughnuts.

Virologists arrived in Halifax on a sunny day in June. Early arrivals took the opportunity to tour Peggy’s Cove or meet for coffee in the Public Gardens. As people arrived at the symposium for registration, they were treated to hot, fresh doughnuts from the Ol’ School Donut Bus (Figure 1).

## 2. 1918 to 2018: One Hundred Years on the Pandemic Road

The symposium opened with a keynote lecture from Dr. David Kelvin (Dalhousie University and Shantou University Medical College) who provided a historical perspective on influenza on the 100th anniversary of the 1918 pandemic. Dr. Kelvin shared anecdotes from the 1918 pandemic and described how factors such as the impact of exposure to past circulating viruses may have influenced pathogenic outcome to the 1918 pandemic virus. He discussed the circumstances of other influenza pandemics of the past two centuries, and the unpredictable evolution of pandemic viruses, whereby some pandemic viruses can enter circulation as an endemic virus, whereas others, such as the 1957 H2N2 virus, can disappear entirely. Lessons learned from the study of these viruses could be applied to prevent or mitigate the impact of another influenza pandemic.

## 3. CRISPR-Cas Systems and Phages: The Ongoing Battle

The 2nd keynote address of the evening was delivered by Dr. Sylvain Moineau (Université Laval) who transitioned from human hosts to bacterial hosts in a fascinating talk about CRISPR-Cas antiviral systems. Bacteria thrive in virus-rich ecosystems by using a combination of antiviral defenses including restriction endonucleases and CRISPR-Cas enzymes that effectively act as bacterial intrinsic and adaptive immune systems, respectively. Dr. Moineau focused on bacterial CRISPR-Cas type II systems that function by archiving short DNA “spacers” derived from invading phage genomes into a CRISPR array in their genome, providing a chronological record of past infections. The CRISPR array is then transcribed into a “guide” RNA that associates with a Cas endonuclease and allows it to cleave incoming foreign viral DNA. Bacterial viruses, known as phage, are not defenseless; they can bypass CRISPR-Cas targeting by mutation or deletion of the CRISPR target in their genome, or by production of anti-CRISPR proteins that subvert the CRISPR-Cas machinery. Dr. Moineau discussed current and potential applications of CRISPR technology and introduced the audience to a wide variety of newly discovered anti-CRISPR proteins that will undoubtedly be the subject of intense investigation for years to come [2,3]. He also described his efforts to bring CRISPR-Cas technology to the undergraduate laboratory curriculum at Université Laval [4].

## 4. New Virus Discovery in Bats: Preparing for Future Zoonotic Infections

The second day of CSV2018 started early for some, with a student-led Fun Run through South End Halifax and Point Pleasant Park. When Fun Run survivors arrived at breakfast, they were greeted by volunteers who gave them seating assignments that maximized potential interactions between PIs and students. Following this, the first scientific session of the day commenced, chaired by Dr. Hugo Soudeyns (Université de Montréal), on “Emerging Viruses”. To lead off, Dr. Vikram Misra (University of Saskatchewan, Saskatoon, SK) presented his studies of bat viruses. In recent years several viruses that appear to cause no overt disease in their natural bat hosts have been transmitted to new mammalian hosts, causing serious and sometimes fatal disease. Dr. Misra’s research program is focused on examining the apparently benign relationship between these viruses and their natural bat hosts, as well as factors that upset this relationship leading to increased viral replication and transmission to new mammalian hosts (recently reviewed in [5]). His team, in a comparison of bat and human host responses to viral infection, demonstrated that while both animals mount antiviral responses, inflammatory responses are diminished in bat cells [6] potentially decreasing viral pathology. Moreover, antiviral responses in bat cells were resistant to viral subversion mechanisms that normally operate in human host cells. The Misra laboratory has identified a new bat coronavirus [7] and a new bat herpesvirus [8] that persistently infect two Canadian bat species with no overt signs of disease. Both viruses infect many, if not most, bats in long-term associations without causing overt disease. The stress of white-nose syndrome, a fungal infection that has decimated some north American bat species, leads to a dramatic increase in corona virus replication in little brown bats, potentially increasing the chances of transmission to other species [9]. Long-term viral persistence followed by stress-induced reactivation could be modeled in tissue-culture where the MERS-coronavirus persistently infected bat cells and the suppression of innate antiviral responses led to increased viral replication [10]. Studying interactions between bat viruses and their natural host may provide key information to prevent future zoonoses.

## 5. Development of a VSV-Based Lassa Virus Vaccine

National Microbiology Laboratory (NML) researchers developed an Ebola vaccine by pseudotyping vesicular stomatitis virus (VSV) with Ebolavirus (EBOV) glycoprotein precursor (GPC), yielding the rVSV-EBOV-GPC vaccine. This vaccine was safe and efficacious in early clinical trials and was deployed in West Africa in 2013 as an emergency measure to quell an Ebola epidemic by ring vaccination (effectively, a phase III clinical trial) [11]. Lassa virus is an arenavirus endemic across much of West Africa. Rodents are the natural host, and zoonotic transmission to humans can cause severe hemorrhagic fevers in 20% of infected individuals. No Lassa virus vaccine is currently available because even though many candidates have performed well in clinical trials, none have yet shown efficacy and safety in humans. NML researchers developed a Lassa fever vaccine using the same VSV platform previously used to create the successful Ebola vaccine by inserting Lassa virus GPC gene into the VSV platform [12]. Dr. Michael Drebot from the Zoonotic Diseases and Special Pathogens Program at the NML summarized the pre-clinical studies conducted to date on the “made-in-Canada” Lassa fever vaccine, which performed well in guinea pig, rhesus and cynomolgus macaque models [13,14,15]. Dr. Drebot also described work from Dr. David Safronetz and other colleagues at the NML who are working on the next steps required to bring this vaccine to clinical trials.

The “Emerging Viruses” session also included presentations from the following trainees: Md Niaz Rahim (University of Manitoba) who discussed a pan-filovirus T-cell vaccine for Ebola and Marburg viruses, Corina Warkentin (University of Ottawa) who discussed roles for kinases in filovirus entry pathways, and Neda Barjesteh (McMaster University) who discussed links between viral infection and Amyotrophic Lateral Sclerosis.

## 6. Early Stages in the Morphogenesis of Herpes Simplex Virus

The next session on “Viral Subversion of Host Cell Processes” opened with a talk on herpesvirus assembly from Dr. Bruce Banfield (Queens University). Herpesviruses replicate in the cell nucleus, where the viral genome is copied into branched concatemers that are trimmed and packaged into newly assembled procapsids. Because these DNA-containing capsids are too large to exit the nucleus via nuclear pores, herpesviruses must transit the nuclear envelope to access the cytoplasm where the final stages of virion maturation occur. This process, called nuclear egress, is achieved by budding of capsids into the inner nuclear membrane yielding an enveloped particle in the perinuclear space. The envelopes of these perinuclear virions then fuse with the outer nuclear membrane releasing DNA-containing capsids into the cytoplasm. Although this fusion mechanism is poorly understood, several groups have partially described the remarkable mechanism of budding, whereby two conserved viral proteins, pUL31 and pUL34, assemble into an oligomeric nuclear egress complex (NEC) on the inner nuclear membrane and facilitate capsid recruitment and budding into the perinuclear space [16,17]. Dr. Banfield described the herpes simplex virus type-2 (HSV-2) NEC complex, which is sufficient to promote nuclear envelope vesiculation and drive nucleoplasmic invagination of the inner nuclear membrane in transfected cells. However, these nuclear envelope perturbations are not normally seen in cells infected with wild-type HSV-2 strains suggesting that these NEC activities are negatively regulated during infection. The capacity of the NEC to drive the formation of long inner nuclear membrane-derived tubules that extend deep into the nucleus suggest that capsid envelopment could, paradoxically, occur deeper in the nucleus rather than at the nuclear periphery where marginalized cellular chromatin and the nuclear lamina create formidable barriers to capsid envelopment. pUL21 is a tegument protein that is required for efficient nuclear egress of HSV-2 capsids [18]. New data from the Banfield lab demonstrated that enveloped capsids accumulate in inner nuclear membrane bound structures deep within the nucleus of cells infected with UL21 mutant viruses suggesting that: 1) pUL21 negatively regulates the inner nuclear membrane invagination activity of the HSV-2 NEC; and 2) that inner nuclear membrane invaginations can serve as sites of capsid envelopment.

## 7. Remodeling Reoviruses for Superior Oncolytic Properties

Mammalian orthoreoviruses selectively replicate in cancer cells and have been extensively studied as oncolytic agents in animal models and human clinical trials [19,20]). Dr. Maya Shmulevitz (University of Alberta) described how knowledge of reovirus molecular genetics can be exploited to create mutant viruses that enter and kill cancer cells more effectively without compromising their selectivity towards transformed cells [21]. For example, while virions bearing the full complement of twelve trimeric σ1 attachment protein complexes on their surfaces are relatively stable in the natural enteric environment, experimentally reducing the number of these attachment proteins on the virion accelerated uncoating and replication in cancer cells and increased oncolytic potency in a mouse melanoma model [22,23]. Dr. Shmulevitz also described how σ1 attachment protein cleavage by breast cancer metalloproteases dramatically lowered reovirus infectivity. Mutagenesis of defined protease cleavage sites on σ1 prevented cleavage and inactivation of reoviruses by breast tumor metalloproteases. These studies suggest that reoviruses are quite amenable to repurposing for superior oncolytic properties.

The “Viral Subversion of Host Cell Processes” session also included presentations from the following trainees: Nichole McMullen (Dalhousie University) who reported the unconventional egress mechanisms of non-enveloped reoviruses, Justine Sitz (Université Laval) who described interactions between a human papillomavirus protein and a host DNA repair-specific E3 ubiquitin ligase, and Quentin Osseman (Université de Montréal) who described interactions between respiratory syncytial virus (RSV) and the host autophagy pathway.

Following this session, the CSV conducted its first face-to-face Business Meeting, where members learned about the state of the society budget and adopted a set of by-laws. CSV symposia and trainee-focused opportunities were discussed, as well as imminent plans for the first open election of a CSV executive from the membership (which was completed in late 2018). Meanwhile, in another theatre, many of the trainee attendees were treated to a Writing Workshop designed by science writer Alan Dove, *PLoS Pathogens* editor Karen Mossman and *PLoS ONE* senior editor Eileen Clancy, who presented strategies to communicate scientific discoveries effectively in writing. Particular attention was paid to making a compelling case for the importance of your research, articulation of a clear take-home message, and considering the audience for your research. Due attention was paid to ethical issues in publishing, and open-access publishing was promoted as a mechanism to maximize the impact of research.

## 8. A Chemical Defense against Phage Infection

The first afternoon session focused on “Viruses of Microbes” was chaired by Dr. Karen Maxwell (University of Toronto). In the first talk of the session, Dr. Maxwell described a new host defense mechanism against viral infection. Bacteria have long been known to defend against bacteriophage infection using restriction endonucleases, cell surface modifications and abortive infection systems, and the past 15 years have seen a rapid expansion of our understanding of diverse CRISPR-Cas systems that provide a form of adaptive immune memory for bacteria [24]. All these systems rely on bacterial proteins or protein/RNA complexes to execute antiviral defense. Dr. Maxwell described the discovery of a new chemical defense against phage infection of the soil-dwelling bacteria Streptomyces. These molecules include anthracyclines that act as DNA-intercalating agents that disrupt the early stages of infection by phages with DNA genomes, perhaps by blocking circularization of the incoming linear DNA [25]. Dr. Maxwell showed that Streptomyces bacteria can secrete anthracyclines into the extracellular environment, which are then taken up by neighboring cells to block viral infection. In this way, these chemicals act as broad-spectrum antivirals that can protect and influence the evolution of bacterial communities.

## 9. Following Phages through Fecal Transplants

Fecal microbiota transplants (FMTs) are increasingly used to treat human intestinal diseases including ulcerative colitis [26] and antibiotic-resistant *Clostridium difficile* infections [27]. However, documenting successful transplant of gut microbiota in FMT recipients remains challenging. FMT inevitably transfer bacteriophages as well [28], but little is known about how transferred phage affect the recipient microbiome. Dr. Alexander Hynes (McMaster University) described studies designed to address this directly by studying crAssphage, the most abundant phage in humans. crAssphage was recently discovered by metagenomic sequencing [29] but its natural bacterial host had not yet been identified. Using PCR and metagenomic analysis, Dr. Hynes’s group tracked crAssphage transfer from phage-positive human donors to human FMT recipients or germ-free mice. Studying the bacteria transferred along with the crAssphage into naïve recipients may provide opportunities to discover the elusive crAssphage host.

The “Viruses of Microbes” session also included presentations from the following trainees: Casey Jones (Dalhousie University) who reported on the state of the gut virome in pediatric Crohn’s Disease [30], Jaclyn McCutcheon (University of Alberta) who presented her discovery of type IV pili as receptors for bacteriophages on Stenotrophomonas maltophilia [31], and Nikhil George (University of Waterloo) who presented his studies of CRISPR-Cas-based warfare between phage and bacteria in a municipal landfill site.

## 10. The Next Generation of Antivirals and Vaccines

Following an afternoon break, Dr. Matthias Götte (University of Alberta) chaired a session on “Antivirals and Vaccines”. This session was kicked off in an electric fashion by four 3-minute thesis-style presentations from trainees. Using only a single slide, Ali Zhang (McMaster University), Briti Saha (University of Ottawa) and Mariel Kleer and Andrea Monjo from Dalhousie University clearly and concisely presented their respective research projects. The general consensus was that future CSV symposia should feature more of these “flash” talks from trainees (and maybe the PIs should be challenged to do the same!). This was followed by a panel discussion about the future of antiviral and vaccine discovery and development, chaired by Dr. Götte and featuring panel members Dr. Marianne Stanford (Vice-President of Research, IMV™), Dr. Marceline Côté (University of Ottawa), Dr. Alyson Kelvin (Dalhousie University) and Dr. David Willer (Global Scientific Affairs Lead – Zoster GSK). This panel provided insight into how new targets are selected and pursued, and the scale of effort required to bring new antivirals and vaccines to market.

## 11. Soapbox Science: Female Scientists Engaging the Public

**Figure 2:**
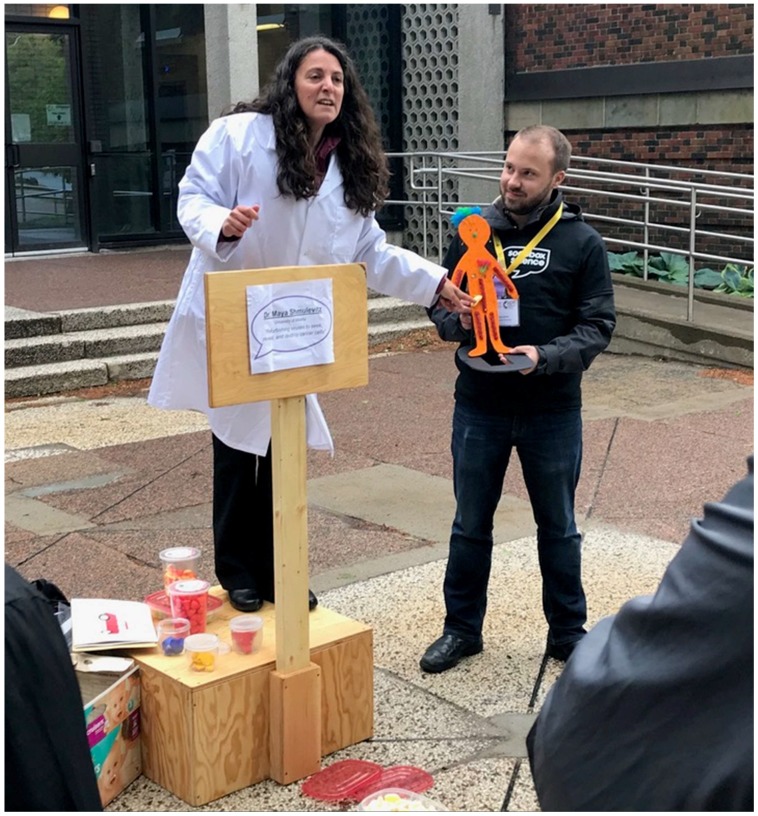
Soapbox Science preview event. Soapbox Science is a science communication event in which female scientists stand on “soapboxes” (see above) and discuss their research with the lay public using simple props. These events are intended to boost the visibility of female scientists and inspire the next generation. In this photo, Dr. Maya Shmulevitz (University of Alberta) is explaining her research program using art supplies and candy; she is illustrating how scientists can transform a virus that CAUSES disease, into a virus that TREATS disease. Volunteer Lucas Jarche (Dalhousie University) is seen here holding a “typical healthy human”, which is composed of many human organs that each have their own unique features (e.g., bones are licorice, heart is a cherry gummy). The audience was asked to make a virus that targets a specific organ, using the corresponding candy. For example, a virus that homes to the heart would have evolved to recognize specific features of the heart/cherry gummy. Additional features of viruses were then added; For example, viruses that cause disease often interfere with immune responses, so candies were added to represent these attributes. Following a discussion of the features of cancer, the audience were instructed to modify their candy viruses so that they will target cancer cells rather than their natural host cells.

Following the “Antivirals and Vaccines” session, everyone had an opportunity to get some fresh air and participate in a preview of Halifax’s first Soapbox Science event, spearheaded by Dalhousie University graduate student Emma Finlayson-Trick. Soapbox Science is an international program that promotes the creation of events that showcase female scientists and provides opportunities for public outreach and science communication. For this preview, Dr. Maya Shmulevitz from the University of Alberta and Drs. Alyson Kelvin and Sarah Wells and PhD student Krysta Coyle from Dalhousie University put on lab coats embossed with the purple Soapbox Science logo and stood on “soapboxes” built by volunteer engineers (Figure 2). They explained their research using simple props to engage and interact with the audience. Of course, CSV2018 attendees were exactly the opposite of the laypeople that Soapbox Science is designed to target, but nevertheless this preview event provided a good example of how scientists can reach out and engage the public. On the Saturday following the symposium, the actual Soapbox Science event launch at the Halifax Seaport Farmers’ Market featured twelve scientists and attracted 600 members of public over 3 h.

## 12. RSV as a Primary Cause of Serious Pneumonia in Children in the Developing World

Following the Soapbox Science preview, the audience returned to the lecture hall for a keynote lecture from Dr. Kate O’Brien, then from Johns Hopkins Bloomberg School of Public Health, who had recently been awarded a prestigious Canada 150 Research Chair to support her recruitment to Dalhousie University (Dr. O’Brien was subsequently recruited by World Health Organization in Geneva, Switzerland, where she is Director of Immunization, Vaccines and Biologicals). Dr. O’Brien’s scientific and policy work is focused on vaccine-preventable illnesses in children and adults. She reported on results from the Pneumonia Etiology Research for Child Health (PERCH) study, which is a collaborative study of populations in seven countries in Africa and Asia designed to determine the etiology of severe pediatric pneumonia in the context of widespread conjugate *Hemophilus influenzae* type B (Hib) and pneumococcal vaccination [32]. The study included over 4000 hospitalized children 0 to 59 months of age, and analysis including molecular and culture methods, chest x-rays and descriptive etiology analysis. The PERCH study identified the frequency of etiologic association of different viruses with severe childhood pneumonia, which notably included RSV, widely recognized as a major cause of childhood mortality worldwide [33]. After the lecture, the audience adjourned to an evening at second poster session, after which, many reunited at a beer garden on Spring Garden Road.

## 13. Flow Virometry: A Novel and Powerful Approach to Decipher Viral Egress

On Friday morning, the first scientific session focused on “Emerging Methods in Virology” was chaired by Dr. Marceline Côté (University of Ottawa) and was led off by Dr. Roger Lippé (Université de Montréal) who described a powerful new technique called flow virometry. Traditionally, flow cytometry has been a very useful approach to study virus infection on a single-cell basis, but viruses themselves have been too small to analyze due to the 0.5 μm resolution of most instruments. Dr. Lippé described how his research group prepares herpesvirus particles for analysis on these instruments by labelling with nucleic acid dyes that can penetrate viral particles or engineering viral particles with fluorescent fusion proteins (including capsid proteins, tegument proteins or glycoproteins) [34,35,36]. This approach enabled characterization of individual HSV-1 particles harvested from different cellular compartments, as well as sorting to >90% purity. Flow virometry also enables the study of the impact of heterogeneity of viral populations on viral fitness and facilitates the analysis by mass spectrometry of highly enriched viral particles isolated along the egress pathway to monitor their maturation. Because flow virometry can be performed on standard flow cytometers and flow sorters available at most institutions, these approaches can be rapidly adopted by others and applied to the study of other large viruses.

## 14. SynViro: Building the Next Generation of Therapeutic Poxvirus Vectors

Low-cost DNA synthesis has catalyzed a new revolution in synthetic biology, enabling efficient manipulation and re-coding of microbial and eukaryotic genomes. Ryan Noyce (University of Alberta) described his efforts to use the principles of synthetic biology to create a synthetic poxvirus vector that could be used for a variety of therapeutic applications. Edward Jenner believed that his variolae vaccinae originated in horses, which has been corroborated by molecular analyses of modern vaccinia virus (VACV) isolates that share common ancestry with horsepox virus (HPXV) [37,38,39] (incidentally, Dr. Noyce told us, this means that the term “vaccination” could be a misnomer; cows may have had nothing to do with it, and the term “equination” may be more accurate [40]). To improve on the safety and efficacy of VACV, Dr. Noyce and colleagues chose to reactivate the ancestral HPXV and use it as a template for safer and more efficacious vaccines and other therapeutics including oncolytic viruses. Dr. Noyce synthesized ten large (10–30 kilobase pair) fragments of DNA based on the published HPXV sequence [38], along with VACV-derived terminal sequences [41]. These sequences were recombined into a 212 kilobase pair live synthetic chimeric (scHPXV) virus in cells infected with Shope fibroma virus (SFV), which served as a helper virus. scHPXV was safe and effective in pre-clinical studies; it produced smaller plaques in cell culture and was less virulent in mice than VACV, but still provided protection against a lethal VACV challenge. Dr. Noyce discussed the technical challenges associated with using synthetic biology as a tool to build new viruses, as well as biosafety and ethical considerations.

The “Emerging Methods in Virology” session also included presentations from the following trainees: Vanessa Meier-Stephenson (University of Calgary and University of Lethbridge) who presented her findings of complex DNA structures in the hepatitis B virus (HBV) genome, Yumiko Komatsu (Kyoto University) who presented her research on Borna disease virus vectors, and Brennan Dirk (Western University) who discussed his efforts to trace multiprotein complexes involved in HIV-1 immune evasion [42,43].

## 15. miR-122-Mediated Protection of the Hepatitis C Virus (HCV) Genome from Pyrophosphatase Activity

Dr. Andrew White (York University) chaired the next session on “RNA in Virus Infection”, which opened with a talk from Dr. Selena Sagan (McGill University). The liver-specific microRNA-122 (miR-122) directly binds to the 5’-end of the hepatitis C virus (HCV) genome and promotes the accumulation of viral RNA in cell culture and animal models, at least in part by protecting the genome from degradation by the host 5’-3’ exonucleases Xrn1 and Xrn2. Because the HCV 5’-triphosphate structure is a molecular pattern expected to be recognized by pattern recognition receptors (PRRs), the Sagan (McGill University) and Wilson (University of Saskatchewan) groups investigated whether miR-122 binding to the HCV genome affects PRR binding and activation of innate antiviral signaling pathways. Dr. Sagan reported that miR-122 does not affect recognition of the HCV genome by protein kinase R (PKR), retinoic acid inducible protein I (RIG-I)-like receptors, or interferon-induced protein with tetratricopeptide repeats (IFITs) 1 and 5 [44]. 5’-triphophate-containing RNAs can be converted to 5’-monophosphate RNA by the action of the cytoplasmic pyrophosphatases DOM3Z and DUSP11; they found that RNA silencing of DOM3Z or DUSP11 rescued RNA accumulation of HCV subgenomic replicons in the absence of miR-122, suggesting that miR-122 normally protects the 5’-triphosphate structure on the viral RNA and prevents pyrophosphatase activity and subsequent degradation by Xrn1/2. Using Selective 2’ Hydroxyl Acylation analyzed by Primer Extension (SHAPE), the Sagan lab is exploring how mutations in the 5’ non-coding region of HCV genomes from patient isolates may help to promote genome stability and relieve HCV’s dependence on miR-122. However, there is evidence to support an additional role for miR-122 in the HCV replication cycle beyond genome stabilization.

## 16. Mechanistic Insight into Influenza Virus Cap-Snatching

Influenza A virus infection delivers eight (−)-sense single-stranded viral RNA (vRNA) genome segments to the cell nucleus, each bound to the tripartite viral RNA-dependent RNA polymerase (RdRp) complex. To initiate transcription, the RdRp complex simultaneously binds to host RNA polymerase II (Pol II) and 5’-cap structures on nascent host pre-mRNAs. Once in position, the polymerase acidic (PA) subunit of the RdRp complex catalyzes endoribonucleolytic cleavage of host pre-mRNAs at a position 10–15 nucleotides downstream of the 5’-cap structure. This process, known as “cap-snatching”, enables priming of viral mRNA synthesis. It remains unclear whether there is some selectivity in this process, or the RdRp simply “snatches” these primers from the most abundant nearby host pre-mRNAs. Dr. Martin Pelchat (University of Ottawa) described deep sequencing studies that support a more nuanced model of cap-snatching that integrates host pre-mRNA abundance with selectivity, whereby each of the eight RdRp:vRNA complexes acquire distinct subsets of host RNAs due to differences in vRNA untranslated region (UTR) sequence [45,46]. Importantly, he reported that host mRNAs associated with antiviral responses are over-represented in the cap-snatched population. This suggests that cap-snatching may indeed play an important role in influenza A virus–host shutoff.

The “RNA in Virus Infection” session also included presentations from the following trainees: Carolyn Robinson (Dalhousie University) who discussed Kaposi’s sarcoma-associated herpesvirus (KSHV) modulation of RNA granules, Eric Pringle (Dalhousie University) who discussed how KSHV mRNAs are translated [47], and Tyler Mrozowich (University of Lethbridge) who presented structural analysis of the Japanese encephalitis virus RNA genome.

## 17. This Week in Virology Podcast

**Figure 3:**
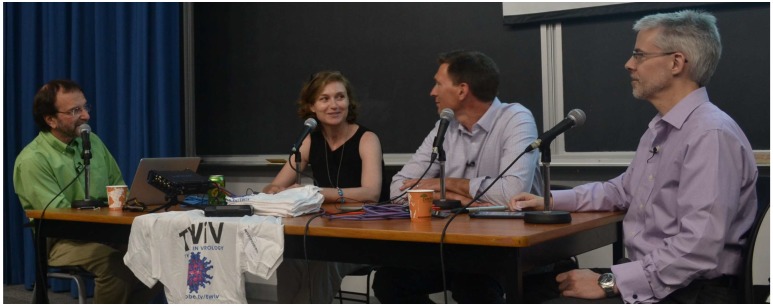
Live-taping of the 499th episode of the This Week in Virology (TWiV) podcast. TWiV was hosted by Dr. Vincent Racaniello (Columbia University, seated far left) and science writer Dr. Alan Dove (seated, far right). Here, Drs. Nathalie Grandvaux and Craig McCormick discuss the Canadian virology community, the origins of CSV, and ideas about the next steps for the society. TWiV 499 also featured interviews with Dr. Ryan Noyce (University of Alberta) and Dr. Kate O’Brien (Johns Hopkins University).

The “This Week in Virology” or “TWiV” podcast was created in September 2008 by Columbia University Professors Vincent Racaniello and Dickson Despommier to discuss science in an informal and informative manner. Ten years later, Dr. Racaniello and TWIV co-host Dr. Alan Dove accepted our invitation to record their 499th podcast at CSV2018. After discussing the weather (as is their custom), the TWiV hosts got down to business with an interview of Drs. Grandvaux and McCormick, who discussed their motivation in founding the CSV, the current research funding climate in Canada, and plans for the new society (Figure 3). They also interviewed Dr. Ryan Noyce from the University of Alberta, who discussed his high-profile synthetic virology (or “SynViro”) research project, in which he used principles of synthetic biology to create a horsepox virus vector that could be used as the basis for new poxvirus vaccines and oncolytic viruses [41,48]. This project had been previously discussed on TWiV 478, entitled “A pox on your horse” [49], and received scrutiny in the scientific and mainstream media due to concerns about ethics and potential “dual-use” of these new poxviruses. Finally, the TWiV team interviewed Dr. Kate O’Brien from Johns Hopkins University, who discussed her global health research in vaccines, and in particular how certain viruses are etiologically linked to severe pneumonia in the developing world. The podcast was entitled “Good virologists go to Halifax” [50].

## 18. Translation Initiation of Alternative Reading Frames by Viral Internal Ribosome Entry Sites

After the TWiV live-taping, our program resumed on the topic of “Viruses of Flora and Fauna”, with Dr. Eric Jan (University of British Columbia) chairing and giving the first talk. Internal ribosome entry sites (IRES) can start translation in alternative reading frames. For example, Dr. Jan’s lab previously showed that the IRES of the honey bee dicistrovirus not only directs translation in the 0 reading frame yielding a polyprotein, but also in the +1 reading frame [51,52]. Dr. Jan reported that the IRES of the related dicistrovirus Cricket paralysis virus (CrPV) also promotes translation in the +1 reading frame, resulting in the translation of a new protein called ORFx [53]. However, unlike the honey bee IRES, a subset of ribosomes recruited to the CrPV IRES can undergo a bypass mechanism to initiate translation downstream at the +1 frame 13th non-AUG codon. Dr. Jan showed that mutagenesis of this downstream +1 frame ORFx attenuates the virus in a Drosophila infection model, suggesting that ORFx plays a role in viral pathogenesis. These studies highlight the diversity of viral translation recoding mechanisms to increase coding capacity.

## 19. Expanding the Repertoire of Plant (+)-Strand RNA Virus Proteases

In the next talk, Hélène Sanfaçon (Summerland Research and Development Centre, Agriculture and Agri-Food Canada) presented evidence for non-canonical proteases encoded by plant viruses. Previously described plant virus proteases are grouped according to conserved catalytic active site residues: cysteine, serine or aspartic acid [54,55]. Dr. Sanfaçon described investigations of viruses of the family *Secoviridae* that constitute a large group of picorna-like viruses [56] and include strawberry mottle virus (SMoV). SMoV has a bipartite genome, with each RNA encoding a large polyprotein. RNA1 encodes a cysteine protease that is related to the canonical 3C protease of picornaviruses and cleaves at multiple locations on polyproteins generated from RNA1 and RNA2 [57]. Dr. Sanfaçon and her team detected an additional proteolytic activity associated with the carboxy-terminal domain of the RNA2 polyprotein [58]. Surprisingly, mutation of conserved serine, cysteine, histidine or aspartic acid residues did not affect this activity. Instead, two glutamic acid residues were shown to be required for polyprotein processing. N-terminal sequencing by Edman degradation method enabled precise mapping of two protease cleavage sites with the consensus sequence P↓xFP. Together, these findings suggest that SMoV polyprotein processing requires two viral proteases, the 3C-like cysteine protease encoded by RNA1, and a novel protease encoded by RNA2 that features two putative glutamic acid residues in the catalytic site. The novel protease domain is only found in the RNA2 polyproteins of two other definite or putative members of the family *Secoviridae*, suggesting that it was recently acquired.

**Figure 4:**
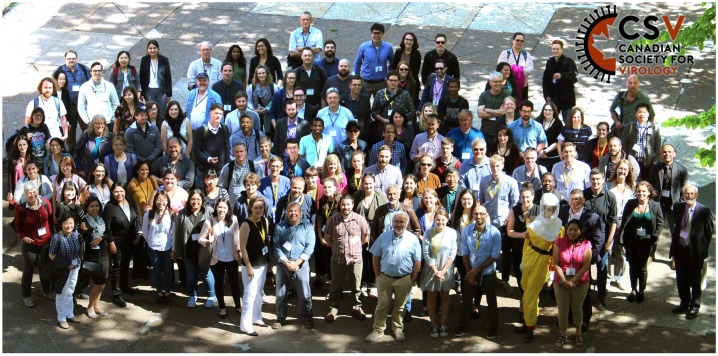
CSV2018 Group Photo.

The “Viruses of Flora and Fauna” session also included presentations from the following trainees: Shuang Yang (University of British Columbia) who described a unique nuclear entry pathway for parvoviruses, Gerard Gaspard (Dalhousie University) who presented mechanistic studies of the reptilian reovirus p14 fusion protein, and Jared Rowell (University of Calgary) who presented studies of cell fate during porcine circovirus-2 infection. During the break following this session, everyone assembled in the courtyard for a group photo (Figure 4).

## 20. Class A Scavenger Receptors in Innate Antiviral Immunity in Lower Vertebrates

Dr. Karen Mossman (McMaster University) chaired the final symposium session on “Antiviral Innate Immunity”, which was led off by her former post-doctoral fellow Dr. Stephanie DeWitte-Orr (Wilfrid Laurier University) who discussed roles for class A scavenger receptors (SR-As) in innate antiviral immunity in lower vertebrates. In mammals the SR-As include five family members (SR-AI, MARCO, SCARA3, SCARA4 and SCARA5) that play diverse roles in immunity, aiding macrophage clearance of pathogens and apoptotic cells, and acting as receptors for immune-stimulating molecules. They have also been shown to serve as virus receptors. Notwithstanding how important these receptors are to host immunity, little is known regarding SR-A function in lower vertebrates. Dr. DeWitte-Orr presented her findings to date regarding SR-As and their functions in fish and frogs. Dr. DeWitte-Orr has identified MARCO, SCARA3, SCARA4 and SCARA5 sequences from rainbow trout [59,60]. These SR-As appear to be functional in rainbow trout, and act as surface receptors for double-stranded RNA (dsRNA), a virus-derived innate immune stimulant [61]. Interestingly, MARCO does not appear to bind dsRNA, a unique finding in vertebrates [59]. SR-As appear to be surface receptors in amphibian cells as well, as Dr. DeWitte-Orr presented her findings regarding SR-A mediated dsRNA responses in the American toad cell line, BufoTad [62]. In addition to function studies, the DeWitte-Orr group has identified a cell line derived from Chinook salmon (CHSE-214) that appears to lack functional SR-As and can be used as a model for SR-A/dsRNA binding [63]. Finally, she presented evidence that SR-As could serve as surface receptors for the ranavirus, frog virus-3 (work described in this Special Issue). Together, these findings provide the first glimpse into the role of SR-As in innate antiviral immunity in lower vertebrates.

## 21. Interferon and Cytokine Responses in Influenza A Virus-Infected Ducks

Continuing with the theme on innate immunity in non-human hosts, Dr. Katharine Magor (University of Alberta) presented her studies of non-pathogenic and pathogenic influenza A virus (IAV) infection of ducks. Dr. Magor shared a photo of herself as a child cradling her pet duck in Yarmouth, Nova Scotia, and explained how this early love of animals influenced her career path. Ducks are the natural IAV reservoir host; the virus infects the gastrointestinal tract and typically does not cause overt disease. However, the emergence of avian H5N1 IAV strains with pandemic potential have disrupted this balance and can cause lethal infection in ducks. To better understand the pathogenic effects of lethal H5N1 infection in ducks, Dr. Magor established an infection model of White Pekin ducks with three similar H5N1 viruses with known differences in pathogenicity and replication rate [64]. Viral replication and host responses were measured by RT-qPCR at different times post-infection. Viral replication peaked and viral load declined in the spleen over time. The ducks rapidly mounted interferon and cytokine responses that peaked on the first day of infection and declined thereafter, with the strongest responses coming from the high-pathogenic viruses. Further characterization of the duck immune responses to lethal and sub-lethal infections could advance our understanding of virus–host interactions in the reservoir host.

The “Antiviral Innate Immunity” session also included presentations from the following trainees: Jiangyi He (Memorial University) who described mechanisms of murine cytomegalovirus immune evasion, Dacquin Kasumba (Université de Montréal) who described roles for DUOX2 enzymes in host responses to respiratory virus infections, and Hannah Stacey (McMaster University) who presented her work on IgA immune complexes in infection and autoimmunity.

## 22. Closing Remarks and Awarding of Prizes

**Figure 5:**
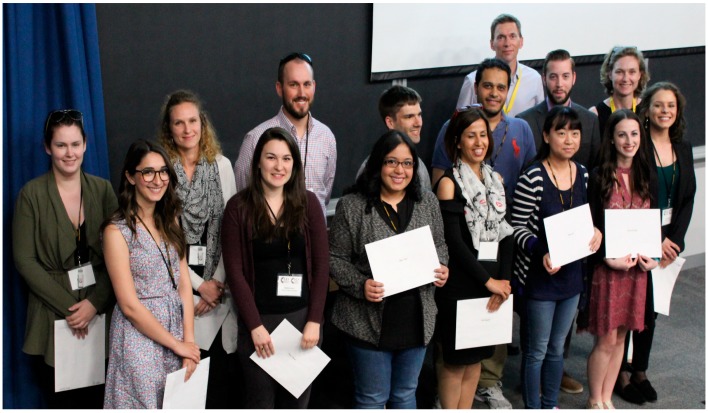
Presentation of travel awards. Each trainee abstract selected for an oral presentation by the CSV2018 Scientific Advisory Committee received a travel award. Front row: Andra Banete (Queen’s University), Natalie Kinloch (Simon Fraser University), Zafrin Islam (Univesity of Toronto), Neda Barjesteh (McMaster University), Zhubing Li (University of Saskatchewan), Hannah Wallace (Memorial University), Danielle Peters (University of Alberta). Back row: Lauren Garnett (National Microbiology Laboratory), Natacha Merindol (Université du Québec à Trois-Rivières), Brennan Dirk (Western University), Bradley Jones (University of British Columbia), Mohamed Abdel-Hakeem (University of Pennsylvania), Tyler Mrozowich (University of Lethbridge), Craig McCormick (Dalhousie University), Nathalie Grandvaux (Université de Montréal). Not pictured: Md Niaz Rahim (National Microbiology Laboratory), Vanessa Meier-Stephenson (University of Lethbridge and University of Calgary).

**Figure 6:**
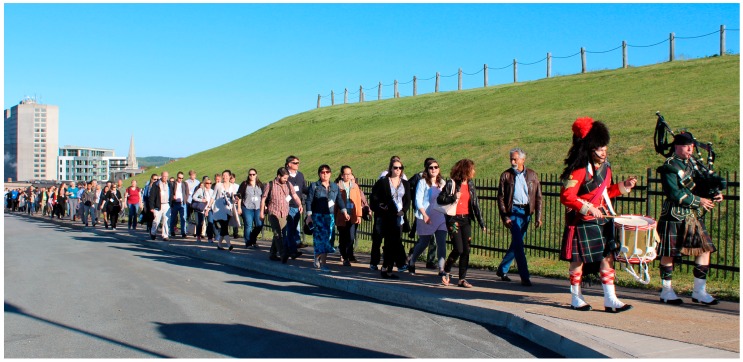
CSV2018 attendees led to the Halifax Citadel National Historic Site. Two members of the 78th Highlanders Regiment ascend Citadel Hill while playing pipe and drum, with symposium attendees in tow.

CSV2018 closed with comments from Dr. Grandvaux thanking sponsors, the scientific advisory committee, and the local organizing committee. Following an ovation for the small army of local volunteers who were instrumental to the success of the symposium, Dr. Grandvaux announced the travel awards (Figure 5). We then gathered outside the lecture theatre where we were met by two members of the 78th Highlanders Pipe Band, who marched us through the streets of Halifax and ascended the slopes of the Halifax Citadel National Historic Site (Figure 6). Inside the walls of the star-shaped fortress, Highlanders conducted historic tours in English and French. Then, the Highlanders gathered us together once again and Dr. McCormick stood on a parapet to announce oral presentation awards to Hannah Stacey from McMaster University and Carolyn Robinson from Dalhousie University, and poster presentation awards to Ali Zhang and Jonathan Mapletoft from McMaster University, Shirley Qiu, Graham Gould Maule and Adrian Pelin from the University of Ottawa, Gillian Singh, Patrick Slaine, Mariel Kleer, Mackenzie Thornbury, and Andrea Monjo from Dalhousie University, Clayton Moore from the University of Guelph and Lauren Garnett from the NML.

**Figure 7:**
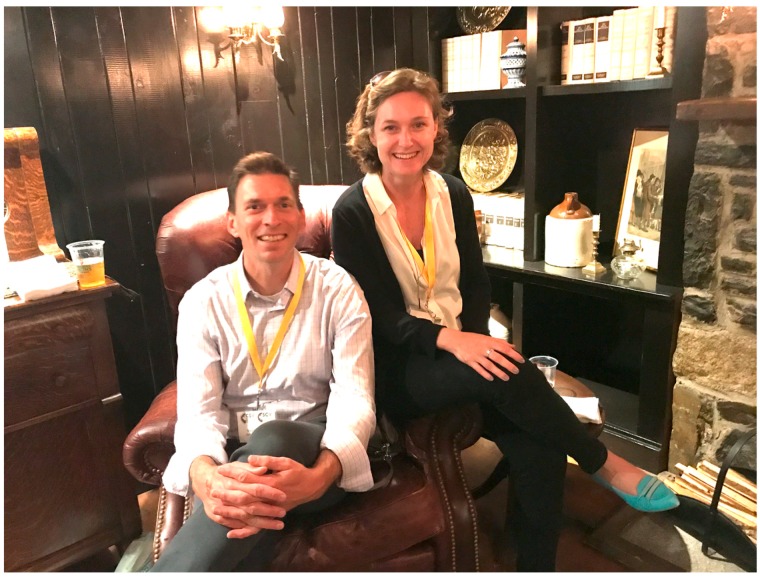
CSV Co-Founders Craig McCormick and Nathalie Grandvaux in Mr. Keith’s Office at the historic Alexander Keith’s Brewery in Halifax, Nova Scotia.

Pipe and Drum sounded once again, and we marched out of the Citadel and down Sackville Street to the historic Alexander Keith’s Brewery for an evening featuring local food, drink, and music. A good time was had by all, including Drs. Grandvaux and McCormick, who could be found relaxing in Mr. Keith’s leather-bound office following a very successful symposium (Figure 7), and perhaps twisting a few arms to encourage colleagues to assume leadership positions and host future CSV symposia.

## References

[1] McCormick C., Grandvaux N. (2017). 1st Workshop of the Canadian Society for Virology. Viruses.

[2] Hynes A.P., Rousseau G.M., Lemay M.L., Horvath P., Romero D.A., Fremaux C., Moineau S. (2017). An anti-CRISPR from a virulent streptococcal phage inhibits Streptococcus pyogenes Cas9. Nat. Microbiol..

[3] Hynes A.P., Rousseau G.M., Agudelo D., Goulet A., Amigues B., Loehr J., Romero D.A., Fremaux C., Horvath P., Doyon Y. (2018). Widespread anti-CRISPR proteins in virulent bacteriophages inhibit a range of Cas9 proteins. Nat. Commun..

[4] Trudel L., Frenette M., Moineau S. (2017). CRISPR-Cas in the laboratory classroom. Nat. Microbiol..

[5] Banerjee A., Kulcsar K., Misra V., Frieman M., Mossman K. (2019). Bats and Coronaviruses. Viruses.

[6] Banerjee A., Rapin N., Bollinger T., Misra V. (2017). Lack of inflammatory gene expression in bats: A unique role for a transcription repressor. Sci. Rep..

[7] Subudhi S., Rapin N., Bollinger T.K., Hill J.E., Donaldson M.E., Davy C.M., Warnecke L., Turner J.M., Kyle C.J., Willis C.K. (2017). A persistently infecting coronavirus in hibernating Myotis lucifugus, the North American little brown bat. J. Gen. Virol..

[8] Subudhi S., Rapin N., Dorville N., Hill J.E., Town J., Willis C.K., Bollinger T.K., Misra V. (2018). Isolation, characterization and prevalence of a novel Gammaherpesvirus in Eptesicus fuscus, the North American big brown bat. Virology.

[9] Davy C.M., Donaldson M.E., Willis C.K., Saville B.J., McGuire L.P., Mayberry H., Wilcox A., Wibbelt G., Misra V., Kyle C.J. (2018). Environmentally persistent pathogens present unique challenges for studies of host-pathogen interactions: Reply to Field (2018). Ecol. Evol..

[10] Banerjee A., Rapin N., Miller M., Griebel P., Zhou Y., Munster V., Misra V. (2016). Generation and Characterization of Eptesicus fuscus (Big brown bat) kidney cell lines immortalized using the Myotis polyomavirus large T-antigen. J. Virol. Methods.

[11] Henao-Restrepo A.M., Longini I.M., Egger M., Dean N.E., Edmunds W.J., Camacho A., Carroll M.W., Doumbia M., Draguez B., Duraffour S. (2015). Efficacy and effectiveness of an rVSV-vectored vaccine expressing Ebola surface glycoprotein: Interim results from the Guinea ring vaccination cluster-randomised trial. Lancet.

[12] Garbutt M., Liebscher R., Wahl-Jensen V., Jones S., Möller P., Wagner R., Volchkov V., Klenk H.D., Feldmann H., Ströher U. (2004). Properties of replication-competent vesicular stomatitis virus vectors expressing glycoproteins of filoviruses and arenaviruses. J. Virol..

[13] Geisbert T.W., Jones S., Fritz E.A., Shurtleff A.C., Geisbert J.B., Liebscher R., Grolla A., Ströher U., Fernando L., Daddario K.M. (2005). Development of a new vaccine for the prevention of Lassa fever. PLoS Med..

[14] Marzi A., Feldmann F., Geisbert T.W., Feldmann H., Safronetz D. (2015). Vesicular stomatitis virus-based vaccines against Lassa and Ebola viruses. Emerg. Infect. Dis..

[15] Safronetz D., Mire C., Rosenke K., Feldmann F., Haddock E., Geisbert T., Feldmann H. (2015). A recombinant vesicular stomatitis virus-based Lassa fever vaccine protects guinea pigs and macaques against challenge with geographically and genetically distinct Lassa viruses. PLoS Negl. Trop. Dis..

[16] Bigalke J.M., Heldwein E.E. (2017). Have NEC Coat, Will Travel: Structural Basis of Membrane Budding During Nuclear Egress in Herpesviruses. Adv. Virus Res..

[17] Hellberg T., Paßvogel L., Schulz K.S., Klupp B.G., Mettenleiter T.C. (2016). Nuclear Egress of Herpesviruses: The Prototypic Vesicular Nucleocytoplasmic Transport. Adv. Virus Res..

[18] Le Sage V., Jung M., Alter J.D., Wills E.G., Johnston S.M., Kawaguchi Y., Baines J.D., Banfield B.W. (2013). The herpes simplex virus 2 UL21 protein is essential for virus propagation. J. Virol..

[19] Coffey M.C., Strong J.E., Forsyth P.A., Lee P.W. (1998). Reovirus therapy of tumors with activated Ras pathway. Science.

[20] Clements D., Helson E., Gujar S.A., Lee P.W. (2014). Reovirus in cancer therapy: An evidence-based review. Oncol. Virother..

[21] Mohamed A., Johnston R.N., Shmulevitz M. (2015). Potential for Improving Potency and Specificity of Reovirus Oncolysis with Next-Generation Reovirus Variants. Viruses.

[22] Shmulevitz M., Gujar S.A., Ahn D.G., Mohamed A., Lee P.W. (2012). Reovirus variants with mutations in genome segments S1 and L2 exhibit enhanced virion infectivity and superior oncolysis. J. Virol..

[23] Mohamed A., Teicher C., Haefliger S., Shmulevitz M. (2015). Reduction of virion-associated σ1 fibers on oncolytic reovirus variants promotes adaptation toward tumorigenic cells. J. Virol..

[24] Makarova K.S., Wolf Y.I., Alkhnbashi O.S., Costa F., Shah S.A., Saunders S.J., Barrangou R., Brouns S.J., Charpentier E., Haft D.H. (2015). An updated evolutionary classification of CRISPR-Cas systems. Nat. Rev. Microbiol..

[25] Kronheim S., Daniel-Ivad M., Duan Z., Hwang S., Wong A.I., Mantel I., Nodwell J.R., Maxwell K.L. (2018). A chemical defence against phage infection. Nature.

[26] Narula N., Kassam Z., Yuan Y., Colombel J.F., Ponsioen C., Reinisch W., Moayyedi P. (2017). Systematic Review and Meta-analysis: Fecal Microbiota Transplantation for Treatment of Active Ulcerative Colitis. Inflamm. Bowel Dis..

[27] Meyers S., Shih J., Neher J.O., Safranek S. (2018). Clinical Inquiries: How effective and safe is fecal microbial transplant in preventing C difficile recurrence. J. Fam. Pract..

[28] Zuo T., Wong S.H., Lam K., Lui R., Cheung K., Tang W., Ching J.Y., Chan P.K., Chan M.C., Wu J.C. (2018). Bacteriophage transfer during faecal microbiota transplantation in Clostridium difficile infection is associated with treatment outcome. Gut.

[29] Dutilh B.E., Cassman N., McNair K., Sanchez S.E., Silva G.G., Boling L., Barr J.J., Speth D.R., Seguritan V., Aziz R.K. (2014). A highly abundant bacteriophage discovered in the unknown sequences of human faecal metagenomes. Nat. Commun..

[30] Douglas G.M., Hansen R., Jones C.M., Dunn K.A., Comeau A.M., Bielawski J.P., Tayler R., El-Omar E.M., Russell R.K., Hold G.L. (2018). Multi-omics differentially classify disease state and treatment outcome in pediatric Crohn’s disease. Microbiome.

[31] McCutcheon J.G., Peters D.L., Dennis J.J. (2018). Identification and Characterization of Type IV Pili as the Cellular Receptor of Broad Host Range Stenotrophomonas maltophilia Bacteriophages DLP1 and DLP2. Viruses.

[32] Levine O.S., O’brien K.L., Deloria-Knoll M., Murdoch D.R., Feikin D.R., DeLuca A.N., Driscoll A.J., Baggett H.C., Brooks W.A., Howie S.R. (2012). The Pneumonia Etiology Research for Child Health Project: A 21st century childhood pneumonia etiology study. Clin. Infect. Dis..

[33] Nair H., Nokes D.J., Gessner B.D., Dherani M., Madhi S.A., Singleton R.J., O’Brien K.L., Roca A., Wright P.F., Bruce N. (2010). Global burden of acute lower respiratory infections due to respiratory syncytial virus in young children: A systematic review and meta-analysis. Lancet.

[34] El Bilali N., Duron J., Gingras D., Lippé R. (2017). Quantitative Evaluation of Protein Heterogeneity within Herpes Simplex Virus 1 Particles. J. Virol..

[35] Lippé R. (2018). Flow Virometry: A Powerful Tool to Functionally Characterize Viruses. J. Virol..

[36] Loret S., El Bilali N., Lippé R. (2012). Analysis of herpes simplex virus type I nuclear particles by flow cytometry. Cytometry A.

[37] Medaglia M.L., Moussatché N., Nitsche A., Dabrowski P.W., Li Y., Damon I.K., Lucas C.G., Arruda L.B., Damaso C.R. (2015). Genomic Analysis, Phenotype, and Virulence of the Historical Brazilian Smallpox Vaccine Strain IOC: Implications for the Origins and Evolutionary Relationships of Vaccinia Virus. J. Virol..

[38] Tulman E.R., Delhon G., Afonso C.L., Lu Z., Zsak L., Sandybaev N.T., Kerembekova U.Z., Zaitsev V.L., Kutish G.F., Rock D.L. (2006). Genome of horsepox virus. J. Virol..

[39] Schrick L., Tausch S.H., Dabrowski P.W., Damaso C.R., Esparza J., Nitsche A. (2017). An Early American Smallpox Vaccine Based on Horsepox. N. Engl. J. Med..

[40] Esparza J., Schrick L., Damaso C.R., Nitsche A. (2017). Equination (inoculation of horsepox): An early alternative to vaccination (inoculation of cowpox) and the potential role of horsepox virus in the origin of the smallpox vaccine. Vaccine.

[41] Noyce R.S., Lederman S., Evans D.H. (2018). Construction of an infectious horsepox virus vaccine from chemically synthesized DNA fragments. PLoS ONE.

[42] Dirk B.S., Pawlak E.N., Johnson A.L., van Nynatten L.R., Jacob R.A., Heit B., Dikeakos J.D. (2016). HIV-1 Nef sequesters MHC-I intracellularly by targeting early stages of endocytosis and recycling. Sci. Rep..

[43] Dirk B.S., van Nynatten L.R., Dikeakos J.D. (2016). Where in the Cell Are You? Probing HIV-1 Host Interactions through Advanced Imaging Techniques. Viruses.

[44] Amador-Cañizares Y., Bernier A., Wilson J.A., Sagan S.M. (2018). miR-122 does not impact recognition of the HCV genome by innate sensors of RNA but rather protects the 5′ end from the cellular pyrophosphatases, DOM3Z and DUSP11. Nucleic Acids Res..

[45] Sikora D., Rocheleau L., Brown E.G., Pelchat M. (2017). Influenza A virus cap-snatches host RNAs based on their abundance early after infection. Virology.

[46] De Vlugt C., Sikora D., Pelchat M. (2018). Insight into Influenza: A Virus Cap-Snatching. Viruses.

[47] Pringle E.S., Robinson C.-A., Crapoulet N., Monjo A.L., Leidal A.M., Lewis S.M., Gaston D., Uniacke J., McCormick C. (2018). mTORC1 activity is dispensable for synthesis of KSHV lytic proteins. bioRxiv.

[48] Noyce R.S., Evans D.H. (2018). Synthetic horsepox viruses and the continuing debate about dual use research. PLoS Pathog..

[49] TWiV 478: A Pox on Your Horse. http://www.microbe.tv/twiv/twiv-478/.

[50] TWiV 499: Good Virologists Go to Halifax. http://www.microbe.tv/twiv/twiv-499/.

[51] Au H.H., Cornilescu G., Mouzakis K.D., Ren Q., Burke J.E., Lee S., Butcher S.E., Jan E. (2015). Global shape mimicry of tRNA within a viral internal ribosome entry site mediates translational reading frame selection. Proc. Natl. Acad. Sci. USA.

[52] Au H.H.T., Elspass V.M., Jan E. (2018). Functional Insights into the Adjacent Stem-Loop in Honey Bee Dicistroviruses That Promotes Internal Ribosome Entry Site-Mediated Translation and Viral Infection. J. Virol.

[53] Kerr C.H., Wang Q.S., Moon K.M., Keatings K., Allan D.W., Foster L.J., Jan E. (2018). IRES-dependent ribosome repositioning directs translation of a +1 overlapping ORF that enhances viral infection. Nucleic Acids Res..

[54] Rodamilans B., Shan H., Pasin F., García J.A. (2018). Plant Viral Proteases: Beyond the Role of Peptide Cutters. Front. Plant Sci..

[55] Mann K.S., Sanfacon H. (2019). Expanding Repertoire of Plant Positive-Strand RNA Virus Proteases. Viruses.

[56] Thompson J.R., Dasgupta I., Fuchs M., Iwanami T., Karasev A.V., Petrzik K., Sanfaçon H., Tzanetakis I., van der Vlugt R., Wetzel T. (2017). ICTV Virus Taxonomy Profile: Secoviridae. J. Gen. Virol..

[57] Mann K.S., Walker M., Sanfaçon H. (2017). Identification of Cleavage Sites Recognized by the 3C-Like Cysteine Protease within the Two Polyproteins of Strawberry Mottle Virus. Front. Microbiol..

[58] Mann K.S., Chisholm J., Sanfaçon H. (2018). Strawberry mottle virus (family Secoviridae, order Picornavirales) encodes a novel glutamic protease to process the RNA2 polyprotein at two cleavage sites. J. Virol..

[59] Poynter S.J., Monjo A.L., Micheli G., DeWitte-Orr S.J. (2017). Scavengers for bacteria: Rainbow trout have two functional variants of MARCO that bind to gram-negative and -positive bacteria. Dev. Comp. Immunol..

[60] Poynter S.J., Leis E.M., DeWitte-Orr S.J. (2018). In vitro transcribed dsRNA limits viral hemorrhagic septicemia virus (VHSV)-IVb infection in a novel fathead minnow (Pimephales promelas) skin cell line. Fish Shellfish Immunol..

[61] Poynter S.J., Weleff J., Soares A.B., DeWitte-Orr S.J. (2015). Class-A scavenger receptor function and expression in the rainbow trout (Oncorhynchus mykiss) epithelial cell lines RTgutGC and RTgill-W1. Fish Shellfish Immunol..

[62] Vo N.T.K., Moore L.C., Leis E., DeWitte-Orr S.J. (2019). Class A scavenger receptors mediate extracellular dsRNA sensing, leading to downstream antiviral gene expression in a novel American toad cell line, BufoTad. Dev. Comp. Immunol..

[63] Monjo A.L., Poynter S.J., DeWitte-Orr S.J. (2017). CHSE-214: A model for studying extracellular dsRNA sensing in vitro. Fish Shellfish Immunol..

[64] Fleming-Canepa X., Aldridge J.R., Canniff L., Kobewka M., Jax E., Webster R.G., Magor K.E. (2019). Duck innate immune responses to high and low pathogenicity H5 avian influenza viruses. Vet. Microbiol..

